# Activation profile of the Atlantic salmon (*Salmo salar*) calcium-sensing receptor (Casr) by selected L-amino acids

**DOI:** 10.1038/s41598-025-97483-5

**Published:** 2025-04-17

**Authors:** Ana S. Gomes, Virginie Gélébart, Rute C. Félix, João C.R. Cardoso, Fabian Zimmermann, Floriana Lai, Deborah M. Power, Ivar Rønnestad

**Affiliations:** 1 Department of Biological Sciences, University of Bergen, Bergen, Norway; 2 Institute of Marine Research, Tromsø, Norway; 3 Centre of Marine Sciences (CCMAR/CIMAR), University of Algarve, Faro, Portugal; 4 International Research Center for Marine Biosciences, Ministry of Science and Technology and National Demonstration Center for Experimental Fisheries Science Education, Shanghai Ocean University, Shanghai, China

**Keywords:** Signaling pathway, G_i_, G_q_, ERK, Nutrient sensing, Teleost, Animal physiology, G protein-coupled receptors

## Abstract

In mammals, the calcium-sensing receptor (CaSR) is involved in nutrient sensing and modulated by several amino acids. In teleosts, sequence homologues of the mammalian CaSR have been described but their function in sensing amino acids remains elusive, including in Atlantic salmon (*Salmo salar*), an important aquaculture species. This study investigated the activation of Atlantic salmon Casr (asCasr)-mediated signaling pathways—G_q_, G_i_, and ERK1/2—by six selected L-amino acids (histidine, tryptophan, phenylalanine, isoleucine, leucine and valine) and by Ca^2+^. Using a Flp-In-HEK293 cell line stably expressing asCasr, we confirmed activation of all three pathways. L-histidine, L-phenylalanine, and L-tryptophan triggered G_i_ signaling independent of Ca²⁺. Notably, no Ca²⁺ concentrations induced G_i_ activation, but IP_1_ production increased in a concentration-dependent manner. L-histidine was the only amino acid to activate the G_q_ pathway without Ca²⁺, and this response was amplified by the presence of Ca²⁺. In the presence of 2.5 mM Ca²⁺, L-phenylalanine and L-tryptophan also activated G_q_ signaling in a concentration-dependent manner. Additionally, in the presence of 10 mM Ca²⁺, L-histidine, L-phenylalanine, and L-tryptophan triggered ERK phosphorylation. These findings establish asCasr as a functional homologue of mammalian CaSR, activated in a concentration-dependent manner by L-amino acids with an aromatic ring.

**Supplementary Information:**

The online version contains supplementary material available at 10.1038/s41598-025-97483-5.

## Introduction

The extracellular calcium-sensing receptor (CaSR) is a member of the class C family of G protein-coupled receptors (GPCRs) and is known for its essential role in regulating serum calcium homeostasis (reviewed in, for e.g.,^1,2^). However, CaSR is a promiscuous receptor, and is activated or modulated by calcium but also by other ligands including L-amino acids (reviewed in^2–4^). The sensing of L-amino acids is vital for activating an appropriate physiological response in whole body protein and amino acid metabolism. In the gastrointestinal tract, amino acids sensing is involved in processes such as the regulation of gastric acid and pancreatic enzyme release (reviewed in^5,6^). In the mammalian intestinal tract, the CaSR is widely expressed in the enteroendocrine I-, K-, and L-^7,8^cells, where it functions as an L-amino acid sensor. The mammalian CaSR is particularly responsive to aromatic L-amino acids such as phenylalanine (L-Phe) and tryptophan (L-Trp), followed by histidine (L-His) in terms of efficacy^9^, and in terms of potency in regulating parathyroid hormone secretion^10^. On the other hand, branched chain amino acids (BCAAs), i.e., valine (L-Val), isoleucine (L-Ile) and leucine (L-Leu), are not effective in modulating the CaSR^9^. In response to specific L-amino acids, the CaSR mediates secretion of regulatory peptide hormones in the gastrointestinal tract, such as cholecystokinin (CCK)^8,11–13^, gastrin^14^, glucose-dependent insulinotropic peptide (GIP), glucagon-like peptide- 1 (GLP- 1), and peptide YY (PYY) secretion^7,12^.

The CaSR mediated regulatory peptide hormone secretion is modulated by its downstream intracellular signaling molecules. These include the activation of G_q_ which results in phospholipase C (PLC) mediated Ca^2+^ mobilization from intracellular stores, the activation of G_i_ which inhibits adenylyl cyclase activity and suppresses cAMP synthesis, and the phosphorylation of several important intracellular protein kinases including the extracellular signal-regulated kinase 1/2 (ERK1/2) and protein kinase C (PKC) (reviewed in^15^). However, the molecular mechanisms involved in the activation of these signaling pathways are not fully understood and are thought to be cell-type-specific, dependent on the G protein type and enzyme expression^16,17^.

Despite the importance of the mechanisms sensing amino acids in the gastrointestinal tract, in teleost fish species the receptors involved in these processes are elusive, as are the mechanisms by which they sense amino acids. This includes the well-studied Atlantic salmon (*Salmo salar*), a major aquaculture species with substantial economic value and where the cost of feed represents a major share of the production expenses^18,19^. Thus, it is surprising that there is not more research on gastrointestinal tract nutrient sensing in farmed species, where such mechanisms are important for the regulation of gastrointestinal function, satiety, and energy homeostasis. Three *casr *transcripts have been identified in the Atlantic salmon^20,21^, *casr1*, *casr2* and *casr3*. At the predicted amino acid sequence level, Atlantic salmon Casr1 and Casr2 share 99.9% identity (only one amino acid residue differs, D257G), while Casr3 shares 89% identity with Casr1 and Casr2, and it is truncated, missing 91 amino acids in the C-terminus, and is suggested to be inactive (see Jury et al.^21^, for details on the three Atlantic salmon Casr sequences). Expression analysis by RT-PCR revealed the presence of *casr *transcripts in the gill, olfactory lamellae, urinary bladder, kidney, intestine, stomach, and brain of Atlantic salmon^21^. Furthermore, the mRNA expression of *casr* in the Atlantic salmon is modulated by salinity, and it has been suggested that the olfactory response to changes in Na^+^, Ca^2+^ and Mg^2+^concentrations occurs via Casr, indicating its involvement in osmoregulation^21^.

In the present study, we developed a robust method to conduct the pharmacological characterization of the Atlantic salmon Casr1 (herein named asCasr), as a first step towards an *in vitro* assay for testing the suitability of dietary ingredients. For validation, Ca^2+^ as well as 3 amino acids (L-Trp, L-Phe and L-His) known to activate the mammalian CaSR and 3 amino acids (the BCAAs, L-Ile, L-Leu and L-Val) known to have little or no effect in modulating this receptor were tested. Because CaSR-mediated signaling is ligand-dependent, we aimed to identify which signaling pathways were modulated by asCasr in response to the selected L-amino acids and Ca^2+^.

## Results

### Expression of Atlantic salmon Casr in Flp-In-HEK293 cell line

**Fig. 1:**
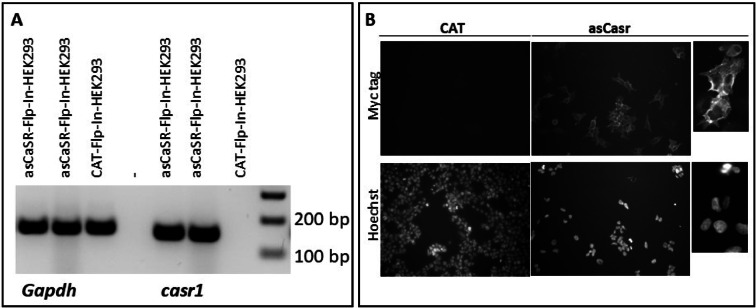
Assessment of stable asCasr-Flp-In-HEK293 cell line. **(A)** Agarose gel of RT-PCR amplicons performed on total RNA extracted from the asCasr-Flp-In-HEK293 and CAT-Flp-In-HEK293 cell lines. PCR products were detected using specific primers for the ubiquitously, and endogenously expressed *Gapdh* and the Atlantic salmon *casr1* gene (please refer to Supplementary Figure S1). **(B)** Immunofluorescent staining of Myc-tagged asCasr protein at the plasma membrane of Flp-In-HEK293 cell (asCasr panel) and pcDNA5/FRT/V5-His/CAT used as a control for pcDNA5/ssCaSR1 (CAT panel). Upper panel: cells were incubated with mouse anti-myc tag antibody followed by staining with Alexa Fluor Plus 488. Lower panel: cells were counterstained with Hoechst 33342. Magnification 10x for left and middle panels of B, and 40x for the right panel.

A stable asCasr-Flp-In-HEK293 cell line was generated, and mRNA expression of the receptor was verified by RT-PCR (Fig. 1A). In addition, since the cell surface expression of the receptor is essential for its activity, the asCasr localization in the cell was confirmed by immunofluorescence using an anti-c-Myc antibody (Fig. 1B).

### G_q_ signaling pathway activation

**Fig. 2:**
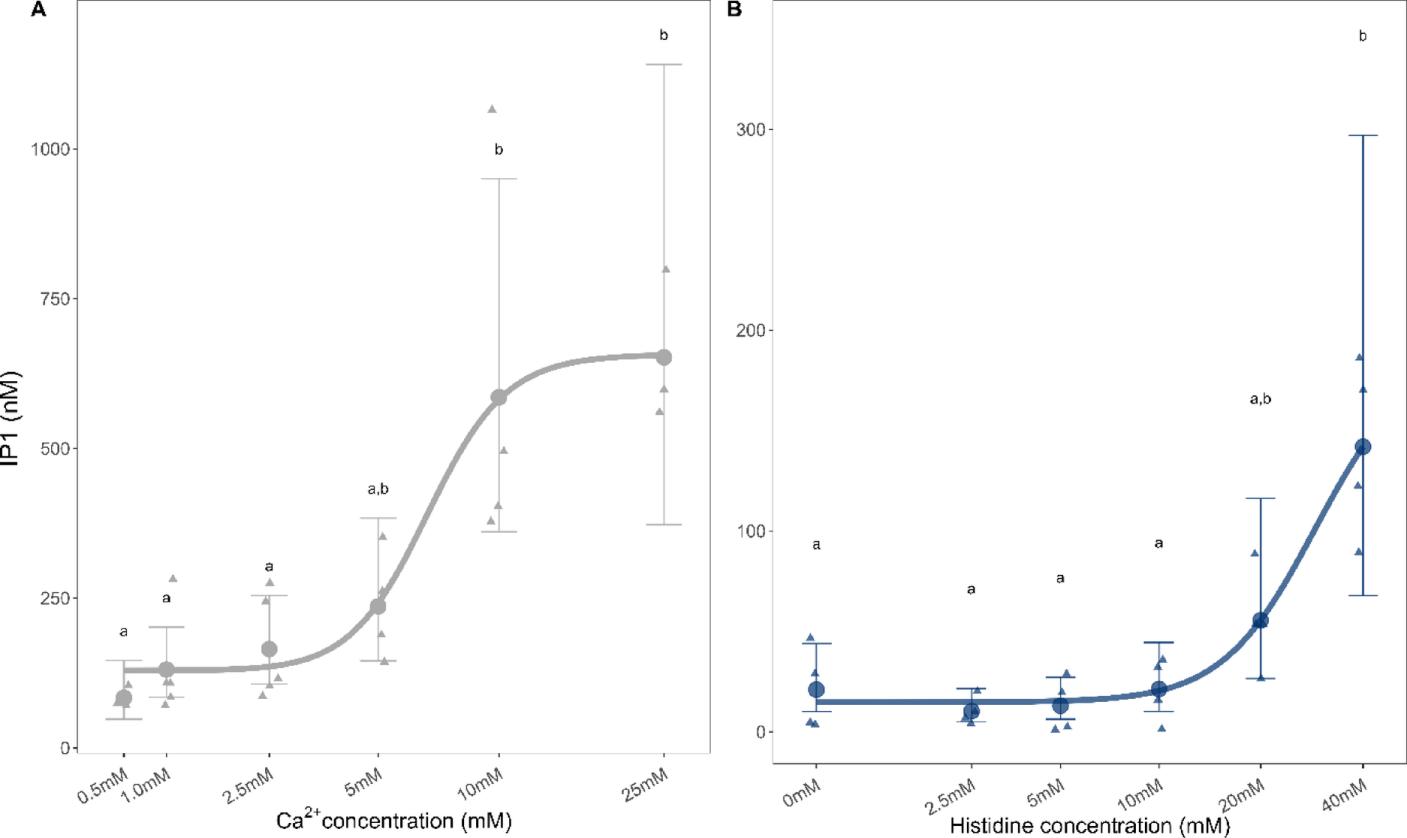
Level of IP_1_ accumulation as a result of G_q_ activation by (**A**) Ca^2+^ and (**B**) histidine (L-His). The concentration-response curve of Ca^2+^ ranged from 0.5 to 25 mM and for L-His ranged from 2.5 to 40 mM with no baseline Ca^2+^ present. The IP_1_ levels were measured in asCasr-Flp-In-HEK293 cells using a HTRF IP-One assay and a Tecan Spark plate reader for (**B**) and a Hidex Sense Plate reader for (**A**). Data are presented as the estimated mean (dots/line) and the 95% confidence intervals (error bars). Raw data for Ca^2+^ comes from three-to-five independent experiments performed in triplicate, and for L-His it comes from four independent experiments performed in triplicate. Significant differences from the basal ligand buffer (represented by 0 mM) and between different L-His concentrations were determined using a general linear model with a gamma distribution (log-link function) followed by Tukey’s post hoc HSD test. Different letters indicate statistically significant differences (*p* < 0.05). For more details, see Supplementary Table S1.

Ca^2+^, L-amino acids without the presence of Ca^2+^, and in the presence of 2.5 mM of Ca^2+^ were tested for their ability to activate the G_q_ pathway in the asCasr-Flp-In-HEK293 cell line. A significant increase in inositol monophosphate (IP_1_) concentration was seen in asCasr-Flp-In-HEK293 for Ca^2+^ in a concentration-dependent manner (Fig. 2A and Supplementary Table S1). Additionally, L-His concentration in the absence of Ca^2+^ also resulted in an increase in IP_1_ production, which was statistically significant for the higher concentrations used, i.e., 40 mM (Fig. 2 and Supplementary Table S1). However, L-His was the only L-amino acid to increase IP_1_ concentration in the absence of Ca^2+^ (Supplementary Fig. S2). When testing L-His in the presence of different concentrations of Ca^2+^ (ranging from 0.5 to 25 mM), a concentration-dependent response was observed for the G_q_ pathway (Supplementary Fig. S3). However, the highest concentration of Ca^2+^ tested, 25 mM, resulted in the opposite trend indicating basically an overload of the system.

**Fig. 3:**
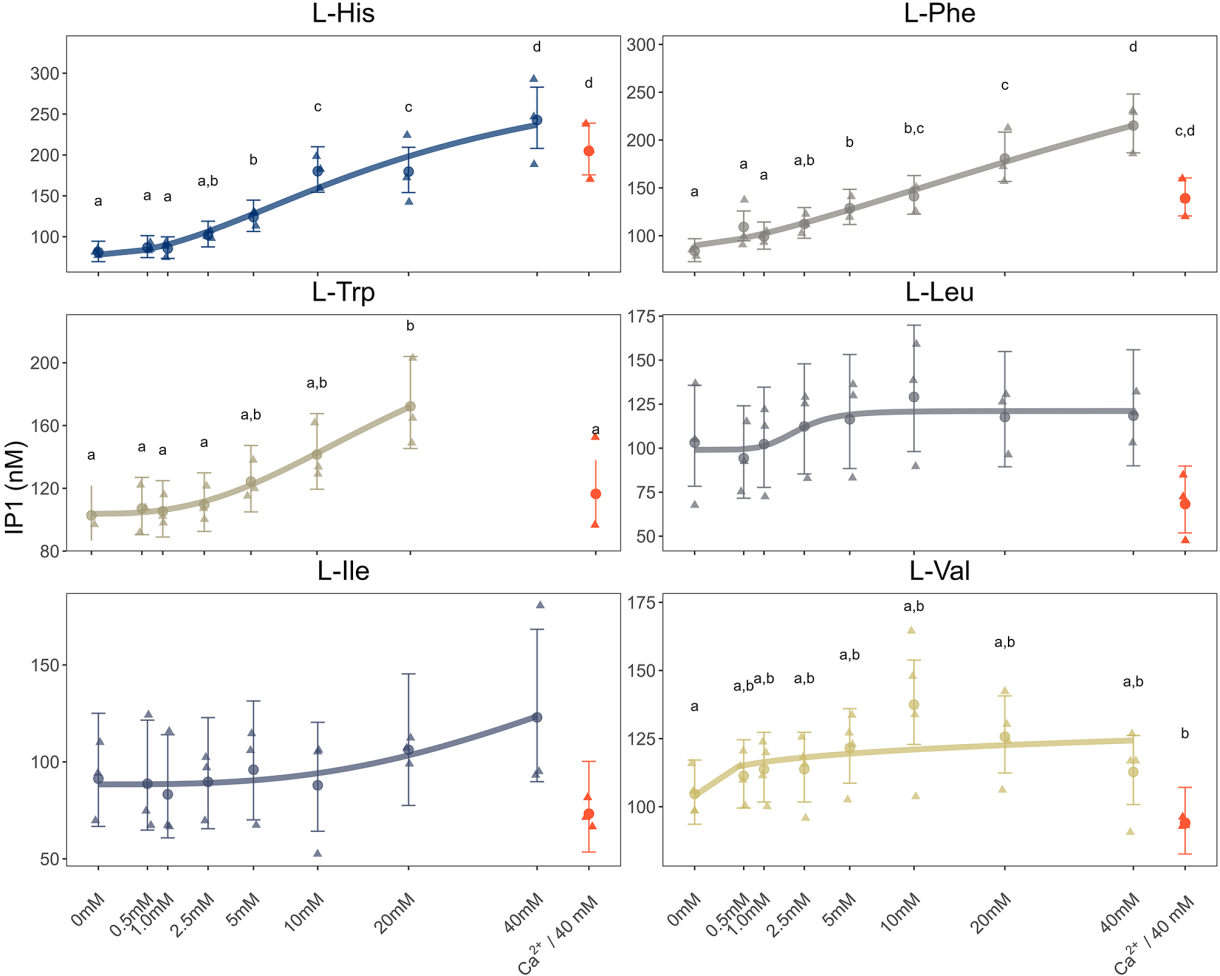
Level of IP_1_ accumulation as a result of G_q_ activation by L-amino acids (concentrations ranging from 0.5 mM to 40 mM, except for L-Trp) in the presence of 2.5 mM of Ca^2+^. The effect of the highest amino acid concentration was also tested under baseline Ca²⁺ conditions (represented in orange). IP_1_ levels were measured in asCasr-Flp-In-HEK293 cells using a HTRF IP-One assay. Data are presented as the estimated mean (dots and lines) and the 95% confidence intervals (error bars), with the raw data of three independent experiments performed in triplicate. Significant differences were determined using a general linear model with a gamma distribution (log-link function) followed by Tukey’s post hoc HSD test. Different letters indicate statistically significant differences (*p* < 0.05). For more details, see Supplementary Table S1.

Based on the Ca^2+^ concentration-response curve, and the L-His concentration-response curves at different Ca^2+^ concentrations, we analyzed the effect of all six L-amino acids in the presence of 2.5 mM of Ca^2+^_,_ a submaximal concentration that was below the maximum possible effect and avoided potential saturation or desensitization of the system. Results showed that L-His, L-Phe and L-Trp in the presence of 2.5 mM of Ca^2+^ significantly affected the IP_1_ concentration (Fig. 3, Supplementary Table 1). L-His exerted effects on the IP_1_ concentration from 10 mM and both L-Phe and L-Trp at 20 mM.

### G_i_ signaling pathway activation

**Fig. 4:**
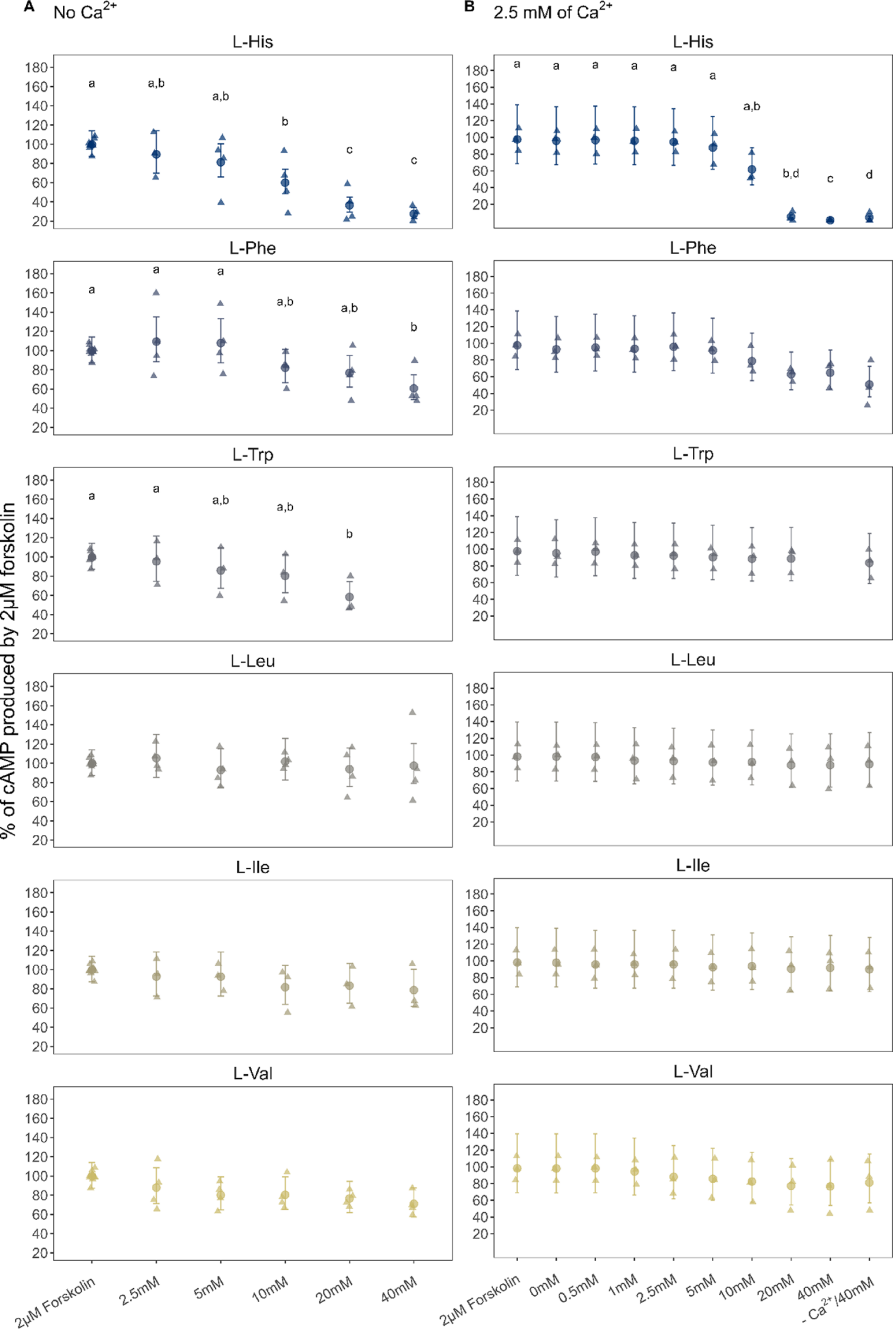
Measurement of cAMP inhibition due to G_i_ activation. Responses to forskolin and histidine (L-His), phenylalanine (L-Phe), tryptophan (L-Trp), leucine (L-Leu), isoleucine (L-Ile) and valine (L-Val) in the absence of Ca^2+^ (**A**) and in the presence of 2.5 mM Ca^2+^ (**B**). The cAMP inhibition was measured in asCasr-Flp-In-HEK293 cells using a HTRF cAMP assay and a Tecan Spark plate reader for (A) and a Hidex Sense Plate reader for (B). Data were normalized to the cAMP production in response to 2 µM forskolin and are shown as the estimated mean (dots) and the 95% confidence intervals (error bars), with the raw data of three to four independent experiments performed in triplicate. Note that -Ca^2+^/40 mM was measured in the presence of baseline Ca^2+^. Significant differences from forskolin (2 µM) and between L-amino acid concentrations were determined using a GLM with gamma distribution (log-link function) followed by a Tukey’s post hoc HSD test. Different letters indicate groups that were significantly different (*p* < 0.05). For more details, see Supplementary Table S2.

To measure the variation of cAMP levels as a result of asCasr activation by Ca^2+^ and L-amino acids, we first inhibited adenyl cyclase by adding Ca^2+^ (concentrations ranging from 0.5 to 25 mM) or L-amino acids (concentrations ranging from 2.5 to 40 mM), and then added 2 µM of forskolin, a diterpene known to activate adenyl cyclase and produce cAMP. The results show that Ca^2+^ did not activate the G_i_ pathway (Supplementary Figure S4 A). Indeed, L-amino acids, particularly L-His, significantly inhibited cAMP levels in both the presence and absence of Ca^2+^ (Fig. 4). These results, in combination with the L-His concentration-response analyses (Supplementary Fig. S4B) reinforced the observation that Ca^2+^ did not influence the levels of cAMP via the G_i_ pathway. From the amino acids tested, L-His, L-Phe, and L-Trp significantly reduced the cAMP level in the asCasr-Flp-In-HEK293 cell line in a concentration-dependent manner (Fig. 4, Supplementary Table S2). In the presence of 2.5 mM of Ca^2+^, only L-His with 20 and 40 mM significantly reduced the cAMP level. The BCAAs, L-Leu, L-Ile, and L-Val, did not have a significant effect on cAMP levels at any of the concentrations tested independent of the presence or absence of Ca^2+^ (Fig. 4). The activation of the G_s_ pathway was also tested in the present of L-amino acids, but no increase in cAMP following the addition of the L-amino acids (data not shown) was observed.

### ERK pathway activation

**Fig. 5:**
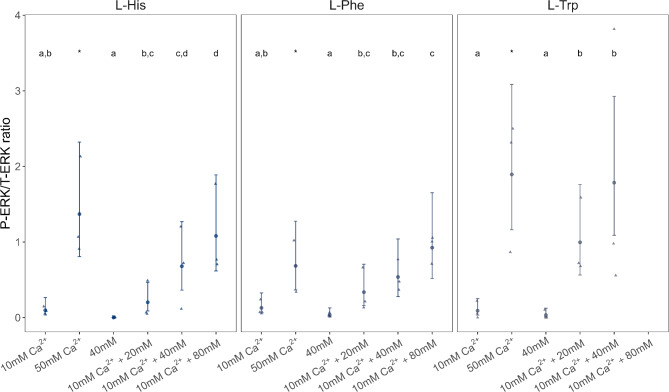
Measurements of ERK activation in asCasr-Flp-In-HEK293 cells after stimulation with histidine (L-His), phenylalanine (L-Phe) and tryptophan (L-Trp) in the presence of 10 mM of Ca^2+^. ERK activation was assessed using antibodies against phosphorylated ERK (P-ERK) and total ERK (T-ERK) after Western blotting. Quantification of the signal on Western blots was performed using the ImageJ program (https://imagej.net/ij/). The P-ERK1/2 response was normalized to the corresponding total ERK1/2 (T-ERK) response, and the P-ERK/T-ERK ratio was calculated as a measure of ERK activation. Data are shown as the estimated mean (dots) and 95% confidence intervals (error bars), with the raw data of three independent experiments. Significant differences from 10 mM Ca^2+^ (concentration necessary for receptor signaling activity) and from 40 mM of amino acid without the 10 mM Ca^2+^ and between different L-amino acid concentrations in the presence of 10 mM of Ca^2+^ were determined using a GLM with tweedie distribution (log-link function) followed by Tukey’s post hoc HSD test. Different letters indicate significant differences, and the “*” indicates significant differences between the two Ca^2+^ concentrations tested, *p* < 0.05 (see Supplementary Table S3 for more detailed information). Representative uncropped images of immunoblots are shown in Supplementary Fig. S5 A-F.

In the absence of Ca^2+^, none of the tested L-amino acids activated the ERK pathway (Fig. 5, Supplementary Fig. S5). Thus, to promote ERK pathway activation, 10 mM Ca^2+^ was added to the cell medium. This Ca²⁺ concentration was below the maximum, as higher concentrations, such as 50 mM, elicited a much stronger response. Among the amino acids tested, L-His, L-Phe and L-Trp, induced an increase in P-ERK levels in asCasr-Flp-In-HEK293 cells (Fig. 5, Supplementary Fig. S5 and Supplementary Table S3). In addition, the activation of the ERK pathway by L-His, L-Phe and L-Trp, was found to be concentration-dependent, i.e., the higher the concentration of each L-amino acid the higher the levels of P-ERK were detected (Fig. 5, Supplementary Table S3).

## Discussion

Previous studies on Casr in fish have focused on identifying tissue distribution profiles, and their function as regulators of Ca^2+ ^homeostasis and salinity sensors^20–25^. In Atlantic salmon, the mRNA and protein of the Casr receptor have been identified in tissues related to osmoregulatory, sensory and neuroendocrine functions, including in the gastrointestinal tract^21^. In addition, Jury et al.^21^ suggested that the location of *casr *on both the apical and basolateral membrane of the intestinal enterocytes indicates an involvement in amino acid sensing, a role that is well described in mammals (reviewed in, for e.g., ^26–28^). Supporting this notion is evidence from rainbow trout (*Oncorhynchus mykiss*) that expression of *casr* mRNA is modulated by amino acids, specifically L-proline, in the proximal intestine^29^. However, to test the hypothesis that asCasr also acts as an amino acid sensing receptor, it is necessary to know whether this receptor is functional and activated by amino acids.

In the present study, a Flp-In-HEK293 cell line that stably expresses the Atlantic salmon Casr was generated. The asCasr is a 7-transmembrane 941-amino acid long GPCR that shares conserved structural features with the human CaSR, including the residues linked to receptor binding and to signaling pathway activation. In addition, the asCasr has a truncated C-terminal region in common with other teleost fishes^23,25^. The asCars-Flp-In-HEK293 cell line was used to test the stimulation by L-amino acids of different intracellular signaling pathways; three of the amino acids chosen in this study are known to stimulate the mammalian CaSR, while the other three have little or no effect on receptor activation^3,4,9,15^. A major limitation when testing receptor activation, including the CaSR, in response to ligands like amino acids or divalent ions, is the presence of the latter in the composition of cell culture media. Establishing a successful stable asCasr-Flp-In-HEK293 cell line that constitutively expresses relatively high levels of the receptor in the plasma membrane circumvented the possible desensitization of the receptor by factors in the culture medium and the need for extensive prewashing of cells^30^, as has previously been demonstrated for other GPCRs by Jacobsen et al.^31^.

Here we investigated to what extent the six selected L-amino acids promoted the activation of asCasr-mediated signaling pathways; G_q_ signaling, G_i _signaling, and ERK1/2, which are known mammalian CaSR-mediated pathways^32^. We confirmed that asCasr activates all three pathways in the asCasr-Flp-In-HEK293 cell line. When analyzing the G_i_ pathway, where the G_i _protein inhibits the enzymatic activity of adenylate cyclase (responsible for the conversion of ATP into the second-messenger cAMP) and results in decreasing levels of cAMP^33^, the three amino acids, L-His, L-Phe, and L-Trp, caused a decline in the cAMP accumulation, independent of the presence of Ca^2+^. None of the tested Ca^2+^ concentrations induced a response in the G_i _signaling pathway, contrary to what has been shown for the mammalian CaSR^15,34^. On the other hand, the addition of Ca^2+^ resulted in a concentration-dependent increase of IP_1_, as reported for the mammalian CaSR^35^. Of the tested amino acids, only L-His triggered a response in asCasr-Flp-In-HEK293 cells when testing the G_q_ signaling pathway in the absence of Ca^2+^. However, in the presence of 2.5 mM of Ca^2+^, the response of L-His was enhanced, and L-Phe and L-Trp also activated the G_q_ pathway in a concentration-dependent manner. This was evident from the increase of IP_1 _production^36,37^. Conigrave et al.^9^ has shown that the amino acid-dependent effects on the intracellular Ca^2+^ (Ca^2+^_i_) mobilization in HEK- 293 cells stably transfected with the human CaSR were observed only when using Ca^2+^ levels above 1.0 mM. Amino acids alone seem to be unable to activate the mammalian CaSR, but they can promote receptor activation in the presence of Ca^2+^. This means that amino acids, in the presence of Ca^2+^, act as a coagonist for CaSR^38,39^. Nonetheless, our results demonstrate that even in the absence of Ca^2+^, L-Trp, L-Phe and L-His activated asCasr-mediated G_i_, while L-His alone activated the G_q_ signaling pathway in the asCars-Flp-In-HEK293 cell line system. However, in the presence of Ca^2+^, G_q_ signaling was further enhanced for L-His, and both L-Trp and L-Phe became activators of this pathway. This suggests that, while the Atlantic salmon CaSR exhibits some level of amino acid-mediated activation independent of Ca^2+^, the presence of Ca^2+^ amplifies the response, particularly for G_q_ pathway.

The presence of Ca^2+^ was essential for the activation of the ERK pathway via asCasr. ERK pathway activation was assessed by detecting the dually phosphorylated serine and threonine residues of the key kinases, ERK1 and ERK2 (P-ERK1/2). We found that in the presence of 10 mM of Ca^2+^, L-His, L-Phe, and L-Trp triggered asCasr-specific ERK phosphorylation. Similarly, L-Phe and L-Trp, but not L-Leu, activated the extracellular Ca^2+^ stimulated ERK1/2 in human CaSR-expressing HEK- 293 cells, but it was comparatively small when compared to the Ca^2+^_i_ effect^40^. The aromatic amino acids L-Phe and L-Trp, and L-His are well established ligands of the mammalian CaSR^3,4,9^. This suggests that the hydrophobic interaction between the aromatic ring of the amino acids and the hydrophobic residues of the CaSR might be important for binding^41^. Our results indicate that the amino acid ligand preference is well-conserved between the Atlantic salmon and the mammalian receptor, as asCasr is also responsive to L-His, L- Phe and L-Trp, which promoted the activation of asCasr-mediated pathways. Furthermore, like the mammalian CaSR^9,42^, BCAAs were less potent activators (or almost inactive) for the asCasr, supporting the notion that the presence of an aromatic ring facilitates ligand-receptor interactions.

The CaSR-mediated gut hormone secretion is regulated by the activation of its downstream signaling molecules^43^. The information obtained in this study and from previous studies, led us to hypothesize that asCasr acts as a nutrient sensor in the gut of Atlantic salmon, and enables specific amino acids to stimulate the release of gut hormones through activation of this receptor. This hypothesis is supported by the following results: (1) *casr *mRNA is present in the gastrointestinal tract of Atlantic salmon^21^; (2) here we show that the L-amino acids L-Phe, L-Trp and L-His and Ca^2+^ activate the asCasr and asCasr-mediated signaling pathways; (3) fish *casr *mRNA in the intestine has been shown to be modulated by amino acids^29^; (4) residues known to be involved in L-amino acid binding are conserved in the asCasr protein sequence (see Supplementary Fig. S6). Although nutrient sensing is essential for survival, surprisingly little is known about receptors (or transporters) involved, especially in fish species. While our results are based on *in vitro* data using a mammalian cell line, the amino acids and Ca^2+^used are within the range reported for the fish intestine^44,45^, and plasma^46^. However, it is important to note that the amino acid profile in the fish gastrointestinal tract vary depending on factors such as diet, nutritional status, and life stage. Additionally, the feed composition and dietary levels of calcium and amino acids can influence the activation of the asCasr receptor in the gastrointestinal tract. Unlike the simplified conditions of our *in vitro* experiments, the gastrointestinal tract will naturally contain a complex mix of nutrients, which may modify receptor activation. Therefore, while these *in vitro* results provide valuable insights into the receptor’s potential function, caution should be exercised when directly translating or comparing them to *in vivo* models, as the *in vivo* environment is more complex and involves additional factors that could influence ligand receptor activation. Nevertheless, understanding the ability to modulate these signaling pathways through diet provides a promising strategy for developing science-based feed formulations that optimize growth, health, and feed efficiency in farmed fish. Overall, our results, along with other recent studies in fish species, suggest that the role of nutrient sensors is broadly conserved between teleosts and mammals^29,47,48^.

In summary, we have successfully generated a stable asCars-Flp-In-HEK293 cell line and validated methods to measure ERK, G_i_ and G_q_-mediated responses. This cell-based assay system provides a valuable tool for screening molecules to study the physiological function of the asCasr and its response to various ligands. Our findings confirm that L-amino acids (L-Phe, L-Trp and L-His) and Ca^2+^ act as ligands for asCasr. Thus, asCasr, like its mammalian homolog, is a promiscuous L-amino acid receptor with preference for amino acids with an aromatic ring. However, different L-amino acids may not be equally effective with respect to all signaling pathways. Notably, only L-His activated the G_q_ pathway in the absence of Ca²⁺, whereas L-Trp and L-Phe required Ca²⁺ for activation, highlighting its role as a coagonist. For the G_i_ pathway, all three amino acids (L-His, L-Phe, and L-Trp) activated signaling both with and without Ca²⁺. However, for the ERK pathway, Ca²⁺ was essential for activation by these three L-amino acids. Amino acids are essential for life, health, and growth, and reliable *in vitro* screening methods for testing amino acids are needed to develop science-based feed formulations for farmed fish.

## Methods

### Materials and reagents

The following materials and reagents, Flp-In™− 293 Cell Line (Cat. No: R75007, lot No: 20167722), Flp-In™ Core System (Cat. No: K601002), Goat anti-Mouse IgG (H + L) Highly Cross-Adsorbed Secondary Antibody, Alexa Fluor Plus 488 (Cat. No: A32723), Hoechst 33342 Solution (20 mM, Cat. No: 62249), Hygromycin B (50 mg/mL, Cat. No: 10687010), polyclonal IgG (H + L) Donkey anti-Rabbit, HRP (Cat. No: A16023), polyclonal IgG (H + L) Rabbit anti-Mouse, HRP (Cat. No: A16160), Lipofectamine™ 2000 Transfection Reagent (Cat. No: 11668 - 019), Myc Tag Monoclonal Antibody (Cat. No: R950 - 25), ProLong™ Gold Antifade Mountant (Cat. No: P10144), Qubit™ Protein Assay Kit (Cat. No: Q33212), Zeocin (100 mg/mL, Cat. No: R25001), Fisherbrand™ Borosilicate Glass Circle Coverslips (Cat. No: 12333138), Antibiotic-Antimycotic (100X, # 15240062), Hanks’ balanced salt solution 10X, no calcium, no magnesium, no phenol red (HBSS 10X, # 14185 - 052), L-glutamine (200 mM, # 25030081), paraformaldehyde 16% (w/v) in aqueous solution methanol-free (Cat. No: 43368.9 L), were obtained from Thermo Fisher Scientific (Waltham, MA, USA). All primes used, agarose (Cat. No: A9539), Bovine serum albumin (Cat. No: A2153), calcium chloride (CaCl_2_, Cat. No: 746495), cOmplete™ Mini Protease Inhibitor Cocktail (Cat. No: 04–693-124 - 001), Dulbecco’s Modified Eagle’s Medium high glucose (DMEM, Cat. No: D6429), dimethyl sulfoxide (DMSO, Cat. No: D5879), DL-Dithiothreitol solution (Cat. No: 43816), fetal bovine serum (FBS, Cat. No: F7524), forskolin (Cat. No: F3917), HEPES (H3375 - 250G), 3-isobutyl- 1-methylxanthine (IBMX, Cat. No: I5879), L-amino acids (LAA21 - 1 KT), lithium chloride (LiCl, Cat. No: L9650), magnesium chloride (MgCl_2_, Cat. No: M2670), phosphate buffered saline 10X (PBS, Cat. No: P5493), Poly-L-lysine hybridomide (P6282 - 5MG), Ponceau S solution (Cat. No: P7170), trypsin-EDTA solution (Cat. No: T3924), Tween- 20 (Cat. No: P9416) were obtained from Sigma Aldrich (St. Louis, MO, USA). The Precision Plus Protein™ WesternC™ Blotting Standards (Cat. No: 1610376), Buffer: 10X Tris/Glycine/SDS Buffer (Cat. No: 1610732), ECL: Clarity Western ECL Substrate (Cat. No: 1705061), Gel: 4–20% Criterion™ TGX Stain-Free™ Protein Gel, 18 well, 30 µl (Cat. No: 5678094), 4x Laemmli Sample Buffer (Cat. No: 1610747) were obtained from BioRad (Hercules, CA, USA). The mouse monoclonal anti-phospho-ERK antibody (Cat. No: 9106 S), and rabbit monoclonal anti-ERK antibody (Cat. No: 4695 S) were obtained from Cell Signalling (Danvers, MA, USA). The cAMP G_i_ kit (Cat. No: 62 AM9PEB), the G_s_ dynamic kit (Cat. No: 62 AM4PEB), and the IP-One G_q_ kit (Cat. No: 62IPAPEB) were obtained from Cisbio (Codolet, France). The plates for the assays, ProxyPlate- 384 Plus, white 384-shallow well microplate (Cat. No: 6008289) were obtained from PerkinElmer (Waltham, MA, USA). The 100 bp DNA Ladder (Cat. No: N3231S), 1 kb DNA Ladder (Cat. No: N3232S), Gel Loading Dye, Purple (6x), no SDS (Cat. No: B7025S), Q5 High Fidelity DNA polymerase (Cat. No: M0491L), and the restriction enzymes MluI-HF (Cat. No: R3198S), NheI-HF (Cat. No: R3131S), and NotI-HF (Cat. No: R3189S) were obtained from New England Biolabs (Ipswich, MA, USA). Cell culture plates, surface: Cell+, flat base, 6 well (Cat. No: 83.3920.300) and 96 well (Cat. No: 83.3924.300) were obtained from Sarstedt (Nümbrecht, Germany), G418 sulfate (50 mg/mL, Cat. No: 30–234-CR) was obtained from Corning (Corning, NY, USA), RIPA lysis buffer 10x (Cat. No: 20–188) was obtained from Merck Millipore (Burlington, MA, USA), and QIAquick Gel Extraction Kit (Cat. No: 28704) was obtained from Qiagen (Hilden, Germany).

### Expression vector constructs

Atlantic salmon *casr1* (Ensemble acc. no. ENSSSAT00000013782.2; GenBank acc. no. NM_001126231.1, see Supplementary Fig. S6 for sequence details) was amplified from Atlantic salmon hindgut using the following forward, 5’- ATGAGATTTTACCTGTATTAC- 3’ and reverse, 5’- CTACTTCATAGAATTCTTTCT- 3’ primers. The Q5 High Fidelity DNA polymerase was used in the PCR reactions and the PCR reaction was carried out in a GeneAmp PCR system 2700 thermocycler (AB Applied Biosystem) with the following conditions: 98 °C for 30 s, 30 cycles of 98 °C for 10 s, 50 °C for 20 s, 72 °C for 90 s, and a final step at 72 °C for 10 min. The PCR product obtained was run in a 1% agarose gel and gel extracted using a QIAquick Gel Extraction Kit, and subsequently cloned into a pcDNA3.1 vector. Atlantic salmon *casr1 *was then transferred to the vector pEGFPN1 kindly provided by Dr Hans Braüner-Osborne (University of Copenhagen, Denmark), containing an mGluR5 signal peptide and c-myc tag^49^. To do so, *casr1* was amplified from the pcDNA3.1-asCasr construct by PCR using a forward primer, 5’-GCGACGCGTTATGGGCCTCATCAG- 3’, containing a MluI restriction site and a reverse primer, 5’-GCGGCGGCCGCCTACTTCATAGAATT- 3’ containing a NotI restriction site. This construct was digested using MluI and NotI restriction enzymes and inserted into the vector pEGFPN1 pre-digested with the same restriction enzymes. The asCasr in the pEGFPN1 construct had a mGluR5 signal peptide and c-myc tag. This construct was transferred from the pEGFPN1-asCasr plasmid to the pcDNA5/FRT vector by digestion with NheI and NotI restriction enzymes. The absence of mutations in all constructs was verified using Sanger DNA sequencing (The DNA Sequencing Lab, University of Bergen, Norway).

### Generation of Flp-In™− 293 cell lines stably expressing Atlantic salmon Casr

Flp-In HEK293 cells were maintained in DMEM media supplemented with 10% (v/v) dialyzed FBS, 2 mM L-glutamine, and 1% antibiotic-antimycotic solution in a humidified atmosphere (95% air and 5% CO_2_). To generate Flp-In-HEK293 cells stably expressing asCasr, cells were co-transfected with a 1:9 mixture of the pcDNA5/FRT/asCasr construct and the Flp recombinase expression plasmid pOG44 using Lipofectamine 2000 according to the manufacturer’s instructions. Twenty-four hours after the transfection, fresh medium was applied, and 48 h after the transfection, fresh medium containing 200 ug/ml Hygromycin B was added to initiate the selection of asCasr-stably expressing cells. After 12 days, the clones were screened for the expression of Atlantic salmon *casr1* and human *Gapdh* by reverse transcription (RT)-PCR (see primers in Table 1) using Q5 High Fidelity DNA polymerase with cDNA synthesized from 500 ng of total RNA in a GeneAmp PCR system 2700 thermocycler (AB Applied Biosystem) with the following conditions: 98 °C for 30 s, 30 cycles of 98 °C for 10 s, 60 °C for 20 s, 72 °C for 20 s, and a final step at 72 °C for 2 min. The amplicons were run on a 2.0% agarose gel, and identity was verified by Sanger DNA sequencing.

**Table 1:** Primer pairs used to amplify Atlantic salmon *casr* and the human *Gapdh* transcripts in the pcDNA5/FRT/asCasr transfected Flp-In HEK293 cells.

Transcript	GenBank Acc. No	Primer sequences (5’à3’)	Amplicon size (bp)
*casr1*	NM_001126231.1	F: GATTCTATGGAATGGATATAATACTGAGG	160
		R: ACTGGCATCTTTGTGATCACTATACTC	
*Gapdh*	NM_001357943.2; NM_001256799.3; NM_001289745.3; NM_001289746.2; NM_002046.7	F: CGAGATCCCTCCAAAATCAA	170
		R: TTCACACCCATGACGAACAT	

### Immunofluorescence

Cells (5 × 10^3^ cells/well) were seeded onto poly-L-lysine coated glass coverslip in 6-well plate. The next day, cells were rinsed with ice-cold 1X PBS and fixed with 4% paraformaldehyde for 10 min at room temperature. Before immunofluorescence staining the slide was blocked with BSA 3% for 1 h at room temperature and then anti-myc-tag antibody (1:500) added and the slides incubated overnight at room temperature in a humidified chamber. The cells were washed with cold 1X PBS three times for 5 min each and incubated with Alexa 488-labeled anti-goat secondary antibody (1:250) at room temperature for 1 h followed by 5 min incubation in Hoechst 33342 solution. The coverslip was mounted using ProLong™ Gold Antifade Mountant. The cells were examined by fluorescence microscopy (Leica DM6000B, Wetzlar, Germany).

### Amino acid and Ca^2+^ solutions

Six L-amino acids, isoleucine (L-Ile), leucine (L-Leu), valine (L-Val), histidine (L-His), phenylalanine (L-Phe), and tryptophan (L-Trp) were prepared at a stock concentration of 100 mM, except for L-Trp for which a 50 mM stock was prepared, in HBSS 1X, HEPES 20 mM. For IP-One G_q_ and cAMP G_i_ assays, the final concentrations of the amino acids tested were 2.5, 5, 10, 20 and 40 mM except for L-Trp where the highest concentration was 20 mM. For the ERK assay, the final concentrations tested for the amino acids were 20, 40 and 80 mM, except for L-Trp where the highest concentration was 40 mM. The concentration of amino acids used in this study was similar to those used in nutrient sensing analyses for the rat CasR^11,12 ^and in fish^29,44^. Ca^2+^ was prepared at a stock concentration of 500 mM. The concentrations of Ca^2+^tested in this study were within the range found in the intestinal fluid of Atlantic salmon (parr: 14.19 ± 0.85 mmol/L; post-smolt: 14.30 ± 1.34 mmol/L)^45^.

### IP-One G_q_ assay

Subconfluent cells were detached from the cell culture dish using trypsin at 37 °C. Then, 4 volumes of DMEM supplemented with 10% (v/v) FBS were added, and cells were centrifuged at 250 g for 5 min. After the cell pellet was resuspended in assay buffer (HBSS 1X, 20 mM HEPES, 1 mg/mL bovine serum albumin, 0.5 mM CaCl_2_, 0.5 mM MgCl_2_ pH 7.4, at 37⁰C) and incubated for 2 h at 37 °C. Cells were finally resuspended at a concentration of 10^7^ cells/ml. Ligands, prepared in ligand buffer (HBSS 1X, HEPES 20 mM, 0.5 mM MgCl_2_, 40 mM LiCl, pH 7.4) and were added (7 µl/well), in triplicate, to a 384-well ProxyPlate. After, 7 µl/well of cell suspension was added to the 384-well ProxyPlate, which was then sealed and incubated at 37 °C for 1 h. After, 3 µl/well of anti- D-myo-inositol monophosphate (IP_1_) cryptate Tb conjugate and 3 µl/well of IP_1_ d2 conjugate were added to the plate, it was then incubated in the dark for 1 h at room temperature. The plate was read on a Hidex Sense Plate reader (Hidex Oy, Turku, Finland) using the following parameters: excitation at 340 nm and measurements of emission at 615 nm and 665 nm. The fluorescence resonance energy transfer ratios (665 nm/615 nm) were converted to IP_1_ concentrations by interpolating values from an IP_1_ standard curve generated from the IP_1_ calibrator provided by the manufacturer.

### cAMP G_i_ assay

Cell suspensions were prepared as described for the IP-One G_q_ assay to achieve a concentration of 10^6^ cells/ml for measurements of G_i_ signaling. The cell suspension (5 µl/well) was added to a 384-well ProxyPlate followed by the addition of 4 µl/well of the ligand solution (ligand prepared in HBSS 1X, 20 mM HEPES, 0.5 mM MgCl_2_, 100 µM IBMX, pH 7.4) in triplicate. The plate was then sealed and incubated at 37 °C for 30 min. Forskolin (2 µM) was added (1 µl/well) as an agonist of the cAMP G_i_ signaling pathway. The plate was sealed and incubated at 37 °C for another 30 min. Two detection solutions (cAMP d2 conjugate and cAMP Eu-cryptate conjugate solutions) were prepared according to the manufacturer’s instructions. 5 µl/well of each conjugate solution was added to the plate, which was then incubated in the dark for 1 h at room temperature. The plate was read on Hidex Sense Plate reader (Hidex OyT, Turku, Finland); excitation was at 340 nm and measurements of emission at 615 and 665 nm. The fluorescence resonance energy transfer ratio (665 nm/615 nm) was determined, and the data was then normalized to the cAMP production elicited by exposure to 2 µM forskolin.

### ERK assay

A total of 3 × 10^6^ cells/well were cultured in a six-well clear culture plate 24 h before the assay. On the day of the assay, cells were washed with 2 ml/well washing buffer (HBSS 1X, 20 mM HEPES, 1 mg/ml bovine serum albumin, pH 7.4) for 2 h at 37 °C. Ligands (L-amino acid and/or Ca^2+^) prepared in ligand buffer (HBSS 1X, 20 mM HEPES, pH 7.4) were added (1 ml/well) to the cells. The plates were incubated for 20 min at 37 °C, as preliminary pilot studies had shown maximum ERK activation 20 min after the addition of Ca^2+^ (data not shown). Subsequently, the cells were washed twice with ice-cold DPBS; 30 µl/well of ice-cold lysis buffer (stock solution: 7 ml of RIPA buffer + 1 protease inhibitor cocktail tablet) was added, and protein extraction was conducted following the manufacturer’s instructions. The protein concentration in each sample was determined using a Qubit protein determination kit.

SDS-PAGE was run using a Criterion™ Cell, and samples were loaded on a 4–20% Criterion™ TGX Stain-Free™ Protein Gel. The gel was run at 130 V for 1 h, and the proteins were subsequently transferred from the gel to a 0.2 μm nitrocellulose membrane using a Trans-Blot Turbo Transfer System (BioRad). The transfer was run at 1 A and 9–10 V for 30 min. Transfer efficiency was checked with Ponceau S staining. Subsequently, the membranes were incubated in blocking solution (5% dry milk (Tine AS) in TBS with 0.2% Tween- 20) for 1 h at room temperature on a tilting table, followed by overnight incubation at 4 °C in the primary antibody (mouse anti-P-ERK 1/2 antibody diluted 1:1000 in 5% dry milk in TBST). The membrane was rinsed in TBST and then incubated for 1 h in secondary antibody (anti-mouse antibody HRP conjugate diluted 1:20,000 in 5% dry milk in TBST) at room temperature on a tilting table. BioRad Clarity Western ECL Substrates were mixed 1:1, and 1 ml of the detection mix was then added to the protein side of the membranes. Imaging of the chemiluminescence was carried out using a ChemiDoc™ Imaging Systems from BioRad. Then the membranes were incubated with total ERK (T-ERK) antibodies (rabbit anti-T-ERK1/2 antibody and anti-rabbit antibody HRP conjugate) and imaged as described above.

Relative quantification of the Western blots was carried out by measuring the intensity of the protein bands using ImageJ Software version 1.54 d (https://imagej.net/ij/)^50^ and by normalizing the P-ERK response with the corresponding T-ERK response.

### Data analysis

All statistical analyses were carried out using R version 4.2 (https://www.r-project.org/)^51^. The IP_1_ concentration-response curve analysis was performed by fitting a four-parameter log-logistic concentration-response model (Eq. 1) using the drm function from the drc package^52^:

1 $$\:f\left(x\right)=\text{c}+\:\frac{\text{d}-\text{c}}{1+\:{e}^{\left(\text{b}\left(\text{log}\left(\text{x}\right)-\text{log}\left(\text{e}\right)\right)\right)}}\:$$

In Eq. 1, **c** is the minimal response, **d** is the maximal response, **b** is the Hill coefficient, **x** is the concentration and **e** is the concentration necessary to elicit a half-maximum response.

A general linear model (GLM) with gamma distribution (log-link function) was used to model the IP_1 _production and the cAMP inhibition in response to different concentration of L-amino acids, while a tweedie distribution (a compound Poisson-gamma with log-link function) was used for ERK activation to account for observations with zero activation. GLMs were implemented with the glmmTMB package^53^. Pairwise comparisons within the groups were performed using a Tukey’s post hoc HSD test with emmeans package^54 ^and ggplot was used to plot graphs^55,56^. Statistical significance was set at *p* < 0.05.

## Electronic supplementary material

Below is the link to the electronic supplementary material.

Supplementary Material 1

## Data Availability

The datasets used and/or analyzed in this study are available from the corresponding author upon reasonable request. The sequences used in this study are publicly available in the GenBank database (Atlantic salmon casr1: NM_001126231.1; human CaSR: P41180; human Gapdh: NM_001357943.2; NM_001256799.3; NM_001289745.3; NM_001289746.2; NM_002046.7).
