# Supplementary material for: Activation profile of the Atlantic salmon (Salmo salar) calcium-sensing receptor (Casr) by selected L-amino acids
Source: Sci Rep. 2025 Apr 17;15:13236. doi: 10.1038/s41598-025-97483-5 (PMC12006508; doi:10.1038/s41598-025-97483-5)
Supplement: Supplementary file 1 — Supplementary Material 1 [file 41598_2025_97483_MOESM1_ESM.docx]

# Supplementary Material

**Activation profile of the Atlantic salmon (*Salmo salar*) calcium-sensing receptor (Casr) by selected L-amino acids**

Ana S. Gomes^a,b^, Virginie Gélébart^a^, Rute C. Felix^c^, João C.R. Cardoso^c^, Fabian Zimmermann^b^, Floriana Lai^a^, Deborah M. Power^c,d^, Ivar Rønnestad^a^

^a^ Department of Biological Sciences, University of Bergen, Bergen, Norway.
^b^ Institute of Marine Research, Tromsø, Norway.
^c^ Centre of Marine Sciences (CCMAR/CIMAR), University of Algarve, Faro, Portugal.

^d^ International Research Center for Marine Biosciences, Ministry of Science and Technology and National Demonstration Center for Experimental Fisheries Science Education, Shanghai Ocean University, Shanghai, China.

Contents

[Expression of Atlantic salmon Casr in Flp-In-HEK293 cell line 2](#_Toc191484446)

[Supplementary Figure S1 2](#_Toc191484447)

[G_q_ signaling pathway activation 3](#_Toc191484448)

[Supplementary Table S1 3](#_Toc191484449)

[Supplementary Figure S2 4](#_Toc191484450)

[Supplementary Figure S3 5](#_Toc191484451)

[G_i_ signaling pathway activation 6](#_Toc191484452)

[Supplementary Table S2 6](#_Toc191484453)

[Supplementary Figure S4 7](#_Toc191484454)

[ERK pathway activation 8](#_Toc191484455)

[Supplementary Table S3 8](#_Toc191484456)

[Supplementary Figure S5 9](#_Toc191484457)

[Multiple alignment of the Atlantic salmon Casr 10](#_Toc191484458)

[Supplementary Figure S6 10](#_Toc191484459)

[References 11](#_Toc191484460)

## Expression of Atlantic salmon Casr in Flp-In-HEK293 cell line


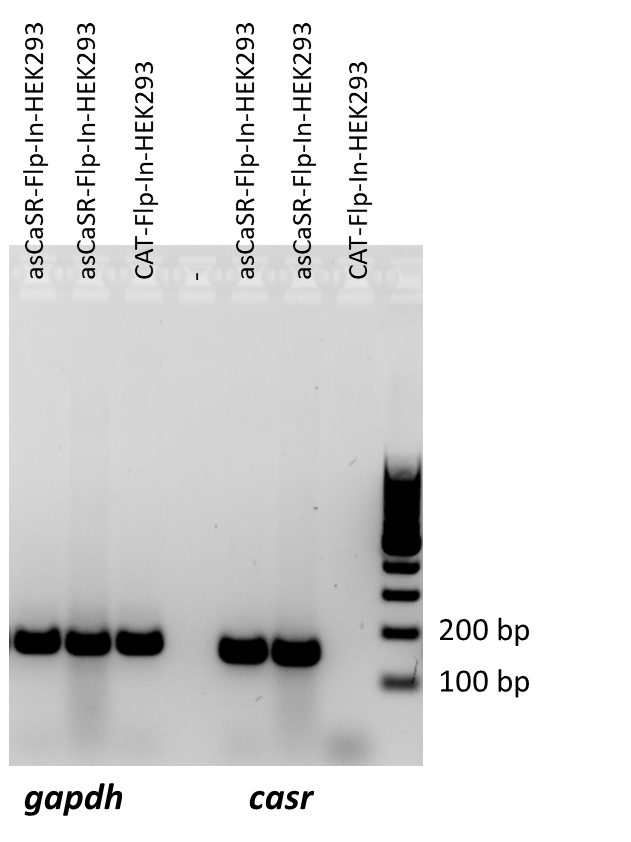


Supplementary Figure S1 Agarose gel of RT-PCR amplicons performed on total RNA extracted from the asCasr-Flp-In-HEK293 and CAT-Flp-In-HEK293 cell lines. PCR products were detected by using specific primers for the ubiquitously, endogenously expressed *Gapdh* and the Atlantic salmon *casr1* gene (see Table 1).

## G_q_ signaling pathway activation

Supplementary Table S1 Results from post-hoc Tukey HSD pairwise test applied to a fitted linear model for IP_1_ accumulation in response to Ca^2+^ and L-amino acids. For each concentration (in mM), the estimated contrast, standard errors (S.E.), degrees of freedom and p-values are shown. Only statistically significant values (p < 0.05) are shown. Note: “no Ca^2+^” indicates baseline Ca^2+^.

|  | **Contrast** | **Estimate** | **S.E.** | **t-ratio** | **p-value** |
| --- | --- | --- | --- | --- | --- |
| **Ca^2+^** | 0.5 mM – 10 mM | -1.9499 | 0.3777 | -5.1631 | 0.0008 |
|  | 0.5 mM – 25 mM | -2.0579 | 0.4037 | -5.0970 | 0.0009 |
|  | 1.0 mM – 10 mM | -1.5001 | 0.3317 | -4.5225 | 0.0031 |
|  | 1.0 mM – 25 mM | -1.6081 | 0.3611 | -4.4531 | 0.0035 |
|  | 2.5 mM – 10 mM | -1.2671 | 0.3317 | -3.8201 | 0.0135 |
|  | 2.5 mM – 25 mM | -1.3751 | 0.3611 | -3.8079 | 0.0138 |
| **L-His** | 0 mM – 40 mM | -1.8984 | 0.5326 | -3.5646 | 0.0229 |
|  | 2.5 mM – 40 mM | -2.6254 | 0.5326 | -4.9295 | 0.0013 |
|  | 5 mM – 40 mM | -2.3940 | 0.5326 | -4.4952 | 0.0032 |
|  | 10 mM – 40 mM | -1.8984 | 0.5326 | -3.5646 | 0.0229 |
| **L-His + 2.5 mM Ca^2+^** | 0 mM – 5 mM | -0.4259 | 0.1108 | -3.8423 | 0.0255 |
|  | 0 mM – 10 mM | -0.7977 | 0.1108 | -7.1969 | <.0001 |
|  | 0 mM – 20 mM | -0.7951 | 0.1108 | -7.1729 | <.0001 |
|  | 0 mM – 40 mM | -1.0958 | 0.1108 | -9.8862 | <.0001 |
|  | 0 mM – 40 mM (no Ca^2+^) | -0.9265 | 0.1108 | -8.3584 | <.0001 |
|  | 0.5 mM – 10 mM | -0.7284 | 0.1108 | -6.5713 | 0.0001 |
|  | 0.5 mM – 20 mM | -0.7257 | 0.1108 | -6.5472 | 0.0001 |
|  | 0.5 mM – 40 mM | -1.0265 | 0.1108 | -9.2605 | <.0001 |
|  | 0.5 mM – 40 mM (no Ca^2+^) | -0.8571 | 0.1108 | -7.7328 | <.0001 |
|  | 1.0 mM – 10 mM | -0.7430 | 0.1108 | -6.7032 | 0.0001 |
|  | 1.0 mM – 20 mM | -0.7403 | 0.1108 | -6.6791 | 0.0001 |
|  | 1.0 mM – 40 mM | -1.0411 | 0.1108 | -9.3924 | <.0001 |
|  | 1.0 mM – 40 mM (no Ca^2+^) | -0.8717 | 0.1108 | -7.8647 | <.0001 |
|  | 2.5 mM – 10 mM | -0.5676 | 0.1108 | -5.1204 | 0.0018 |
|  | 2.5 mM – 20 mM | -0.5649 | 0.1108 | -5.0963 | 0.0019 |
|  | 2.5 mM – 40 mM | -0.8656 | 0.1108 | -7.8096 | <.0001 |
|  | 2.5 mM – 40 mM (no Ca^2+^) | -0.6963 | 0.1108 | -6.2819 | 0.0002 |
|  | 5.0 mM – 40 mM | -0.6699 | 0.1108 | -6.0439 | 0.0003 |
|  | 5.0 mM – 40 mM (no Ca^2+^) | -0.5006 | 0.1108 | -4.5161 | 0.0064 |
| **L-Phe + 2.5 mM Ca^2+^** | 0 mM – 5 mM | -0.4272 | 0.1027 | -4.1587 | 0.0134 |
|  | 0 mM – 10 mM | -0.5198 | 0.1027 | -5.0595 | 0.0021 |
|  | 0 mM – 20 mM | -0.7656 | 0.1027 | -7.4524 | <.0001 |
|  | 0 mM – 40 mM | -0.9404 | 0.1027 | -9.1535 | <.0001 |
|  | 0 mM – 40 mM (no Ca^2+^) | -0.5041 | 0.1027 | -4.9067 | 0.0028 |
|  | 0.5 mM – 20 mM | -0.5040 | 0.1027 | -4.9057 | 0.0028 |
|  | 0.5 mM – 40 mM | -0.6787 | 0.1027 | -6.6068 | 0.0001 |
|  | 1.0 mM – 20 mM | -0.6001 | 0.1027 | -5.8415 | 0.0004 |
|  | 1.0 mM – 40 mM | -0.7749 | 0.1027 | -7.5427 | <.0001 |
|  | 2.5 mM – 20 mM | -0.4757 | 0.1027 | -4.6306 | 0.0050 |
|  | 2.5 mM – 40 mM | -0.6505 | 0.1027 | -6.3317 | 0.0002 |
|  | 5.0 mM – 40 mM | -0.5131 | 0.1027 | -4.9948 | 0.0024 |
|  | 10 mM – 40 mM | -0.4206 | 0.1027 | -4.0940 | 0.0153 |
|  | 40 mM – 40 mM (no Ca^2+^) | 0.4363 | 0.1027 | 4.2469 | 0.0112 |
| **L-Trp + 2.5 mM Ca^2+^** | 0 mM – 20 mM | -0.5165 | 0.1222 | -4.2255 | 0.0115 |
|  | 0.5 mM – 20 mM | -0.4742 | 0.1222 | -3.8799 | 0.0226 |
|  | 1.0 mM – 20 mM | -0.4910 | 0.1222 | -4.0171 | 0.0173 |
|  | 2.5 mM – 20 mM | -0.4517 | 0.1222 | -3.6954 | 0.0322 |
| **L-Val + 2.5 mM Ca^2+^** | 10 mM – 40 mM (no Ca^2+^) | 0.3791 | 0.0875 | 4.3327 | 0.0052 |


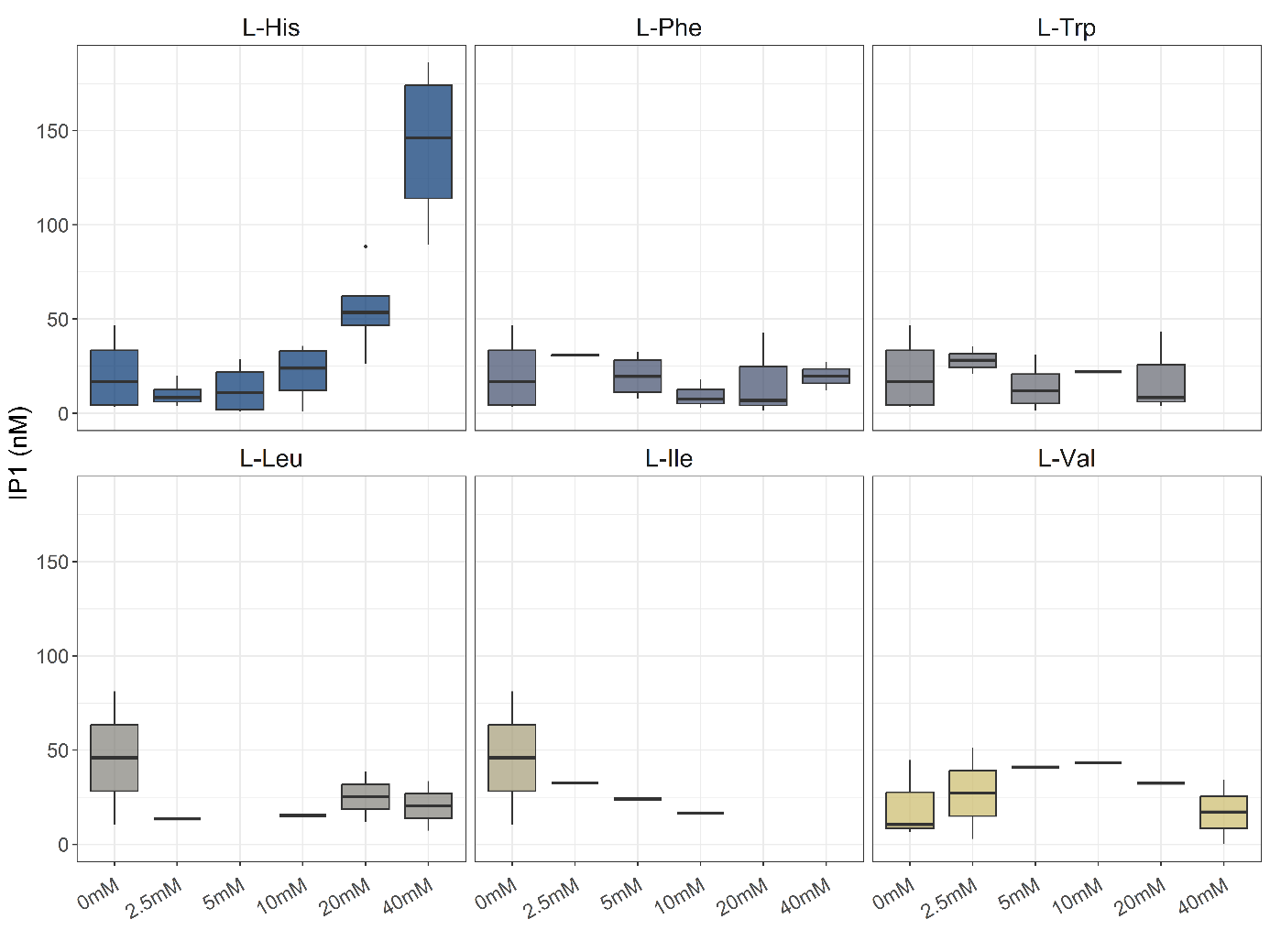


Supplementary Figure S2 Measurements of IP_1_ accumulation as a result of G_q_ signaling pathway activation. Responses to the L-amino acids measured in asCasr-Flp-In-HEK293 cells using the HTRF IP-One assay. The line in the boxplot indicates the median and boxes the 1^st^ to 3^rd^ quartiles, whiskers mark variation outside 1^st^ and 3^rd^ quartiles and dots the outliers of three to four independent experiments performed in triplicate.


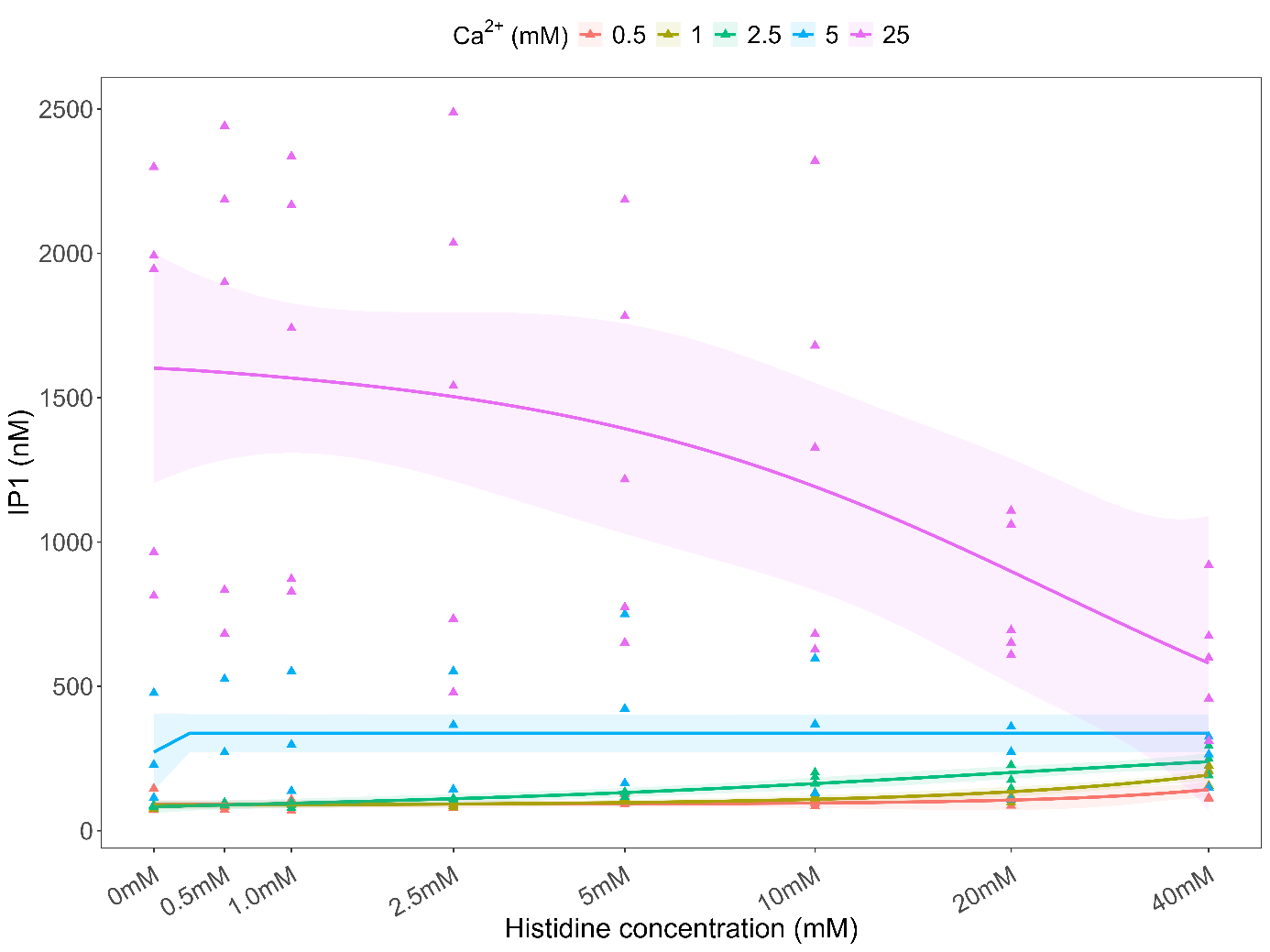


Supplementary Figure S3 Measurements of IP_1_ accumulation as a result of G_q_ signaling pathway activation. Responses to the histidine (L-His) concentrations (0.5 to 40 mM) in the presence of Ca^2+^ (concentration ranging from 0.5 to 5 mM) in asCasr-Flp-In-HEK293 cells using the HTRF IP-One assay. Concentration-response curves are represented by the solid lines and the 95% confidence interval (shaded area) is indicated. The raw data of four to six independent experiments performed in triplicate are indicated by triangles.

## G_i_ signaling pathway activation

Supplementary Table S2 Results from post-hoc Tukey HSD pairwise test applied to a fitted linear model for cAMP inhibition in response to L-amino acids. For each amino acid concentration (in mM), the estimated contrast, standard errors (S.E.), degrees of freedom and p-values are shown. Only statistically significant values (p < 0.05) are shown. Note: “no Ca^2+^” indicates baseline Ca^2+^.

|  | **Contrast** | **Estimate** | **S.E.** | **t-ratio** | **p-value** |
| --- | --- | --- | --- | --- | --- |
| **L-His** | Forskolin – 10 mM | 0.5087 | 0.1277 | 3.9823 | 0.0015 |
|  | Forskolin – 20 mM | 1.0072 | 0.1277 | 7.8848 | <.0001 |
|  | Forskolin –40mM | 1.2897 | 0.1277 | 10.0956 | <.0001 |
|  | 2.5 mM – 20 mM | 0.8980 | 0.1649 | 5.4454 | <.0001 |
|  | 2.5 mM – 40 mM | 1.1805 | 0.1649 | 7.1580 | <.0001 |
|  | 5 mM – 20 mM | 0.8021 | 0.1527 | 5.2533 | <.0001 |
|  | 5 mM – 40 mM | 1.0845 | 0.1527 | 7.1031 | <.0001 |
|  | 10 mM – 20 mM | 0.4985 | 0.1527 | 3.2651 | 0.0172 |
|  | 10 mM – 40 mM | 0.7809 | 0.1527 | 5.1148 | <.0001 |
| **L-Phe** | Forskolin – 40 mM | 0.4971 | 0.1277 | 3.8918 | 0.0021 |
|  | 2.5 mM – 40 mM | 0.5894 | 0.1527 | 3.8602 | 0.0024 |
|  | 5 mM – 40 mM | 0.5755 | 0.1527 | 3.7696 | 0.0033 |
| **L-Trp** | Forskolin – 20 mM | 0.5370 | 0.1421 | 3.7777 | 0.0022 |
|  | 2.5 mM – 20 mM | 0.4916 | 0.1763 | 2.7884 | 0.0472 |
| **L-His + 2.5 mM Ca^2+^** | Forskolin – 20 mM | 2.9338 | 0.2543 | 11.5356 | <.0001 |
|  | Forskolin –40mM | 4.6052 | 0.2543 | 18.1071 | <.0001 |
|  | Forskolin –40mM (no Ca^2+^) | 3.1502 | 0.2543 | 12.3864 | <.0001 |
|  | 0 mM – 20 mM | 2.9166 | 0.2543 | 11.4679 | <.0001 |
|  | 0 mM – 40 mM | 4.5880 | 0.2543 | 18.0394 | <.0001 |
|  | 0 mM –40mM (no Ca^2+^) | 3.1330 | 0.2543 | 12.3187 | <.0001 |
|  | 0.5 mM – 20 mM | 2.9244 | 0.2543 | 11.4983 | <.0001 |
|  | 0.5 mM – 40 mM | 4.5957 | 0.2543 | 18.0699 | <.0001 |
|  | 0.5 mM –40mM (no Ca^2+^) | 3.1408 | 0.2543 | 12.3492 | <.0001 |
|  | 1.0 mM – 20 mM | 2.9164 | 0.2543 | 11.4670 | <.0001 |
|  | 1.0 mM – 40 mM | 4.5877 | 0.2543 | 18.0385 | <.0001 |
|  | 1.0 mM –40mM (no Ca^2+^) | 3.1328 | 0.2543 | 12.3178 | <.0001 |
|  | 2.5 mM – 20 mM | 2.9024 | 0.2543 | 11.4118 | <.0001 |
|  | 2.5 mM – 40 mM | 4.5737 | 0.2543 | 17.9833 | <.0001 |
|  | 2.5 mM –40mM (no Ca^2+^) | 3.1188 | 0.2543 | 12.2626 | <.0001 |
|  | 5.0 mM – 20 mM | 2.8279 | 0.2543 | 11.1192 | <.0001 |
|  | 5.0 mM – 40 mM | 4.4993 | 0.2543 | 17.6907 | <.0001 |
|  | 5.0 mM –40mM (no Ca^2+^) | 3.0443 | 0.2543 | 11.9700 | <.0001 |
|  | 10 mM – 20 mM | 2.4758 | 0.2543 | 9.7345 | <.0001 |
|  | 10 mM – 40 mM | 4.1471 | 0.2543 | 16.3061 | <.0001 |
|  | 10 mM –40mM (no Ca^2+^) | 2.6922 | 0.2543 | 10.5853 | <.0001 |
|  | 20 mM – 40 mM | 1.6713 | 0.2543 | 6.5715 | <.0001 |
|  | 40 mM –40mM (no Ca^2+^) | -1.4549 | 0.2543 | -5.7207 | <.0001 |


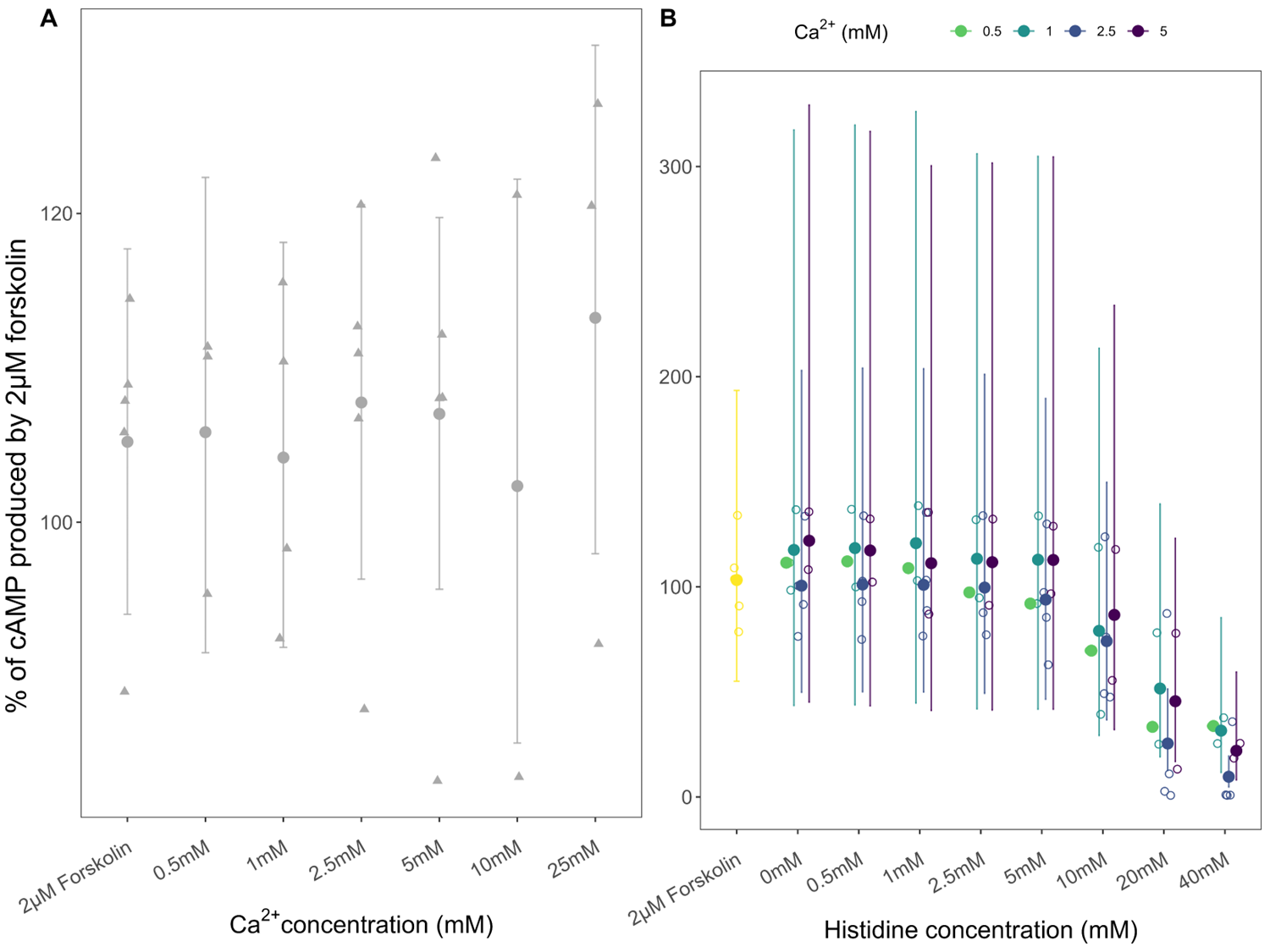


Supplementary Figure S4 Measurement of cAMP inhibition due to G_i_ activation. Responses to Ca^2+^ and histidine (L-His) in the presence of Ca^2+^ (concentration ranging from 0.5 to 5 mM) were measured in asCasr-Flp-In-HEK293 cells using a HTRF cAMP assay. Data is normalized to the cAMP production in response to 2 µM forskolin and is shown as the estimated mean (dots) and the 95% confidence intervals (error bars), with the raw data from one to four independent experiments performed in triplicate.

## ERK pathway activation

Supplementary Table S3 Results from a post-hoc Tukey HSD pairwise test applied to a fitted linear model for ERK pathway activation in response to L-amino acids. For each amino acid concentration (in mM), as well as for the divalent ion calcium (Ca^2+^) concentrations, the estimated contrast, standard errors (S.E.), z-ratio and p-values are shown. Only statistically significant values (p < 0.05) are shown.

|  | **Contrast** | **Estimate** | **S.E.** | **z-ratio** | **p-value** |
| --- | --- | --- | --- | --- | --- |
| **L-His** | 10 mM Ca^2+^ – 50 mM Ca^2+^ | -2.6434 | 0.5758 | -4.5906 | 0.0001 |
|  | 10 mM Ca^2+^ – 10 mM Ca^2+^ + 40 mM | -1.9427 | 0.6006 | -3.2348 | 0.0154 |
|  | 10 mM Ca^2+^ – 10 mM Ca^2+^ + 80 mM | -2.4060 | 0.5834 | -4.1241 | 0.0005 |
|  | 50 mM Ca^2+^ – 40 mM | 6.6309 | 1.3584 | 4.8812 | <.0001 |
|  | 50 mM Ca^2+^ – 10 mM Ca^2+^ + 20 mM | 1.9099 | 0.5043 | 3.7869 | 0.0021 |
|  | 40 mM – 10 mM Ca^2+^ + 20 mM | -4.7210 | 1.3982 | -3.3766 | 0.0096 |
|  | 40 mM – 10 mM Ca^2+^ + 40 mM | -5.9302 | 1.3691 | -4.3314 | 0.0002 |
|  | 40 mM – 10 mM Ca^2+^ + 80 mM | -6.3935 | 1.3617 | -4.6953 | <.0001 |
|  | 10 mM Ca^2+^ + 20 mM – 10 mM Ca^2+^ + 80 mM | -1.6725 | 0.5130 | -3.2604 | 0.0142 |
| **L-Phe** | 10 mM Ca^2+^ – 50 mM Ca^2+^ | -1.6719 | 0.5727 | -2.9193 | 0.0410 |
|  | 10 mM Ca^2+^ – 10 mM Ca^2+^ + 80 mM | -1.9739 | 0.5606 | -3.5211 | 0.0057 |
|  | 50 mM Ca^2+^ – 40 mM | 2.9408 | 0.7207 | 4.0807 | 0.0006 |
|  | 40 mM – 10 mM Ca^2+^ + 20 mM | -2.2340 | 0.7487 | -2.9840 | 0.0339 |
|  | 40 mM – 10 mM Ca^2+^ + 40 mM | -2.7002 | 0.7293 | -3.7027 | 0.0029 |
|  | 40 mM – 10 mM Ca^2+^ + 80 mM | -3.2428 | 0.7111 | -4.5604 | 0.0001 |
| **L-Trp** | 10 mM Ca^2+^ – 50 mM Ca^2+^ | -3.0234 | 0.5729 | -5.2773 | <.0001 |
|  | 10 mM Ca^2+^ – 10 mM Ca^2+^ + 20 mM | -2.3812 | 0.5922 | -4.0208 | 0.0006 |
|  | 10 mM Ca^2+^ – 10 mM Ca^2+^ + 40 mM | -2.9645 | 0.5745 | -5.1603 | <.0001 |
|  | 50 mM Ca^2+^ – 40 mM | 4.0001 | 0.6989 | 5.7236 | <.0001 |
|  | 40 mM – 10 mM Ca^2+^ + 20 mM | -3.3579 | 0.7148 | -4.6977 | <.0001 |
|  | 40 mM – 10 mM Ca^2+^ + 40 mM | -3.9412 | 0.7002 | -5.6290 | <.0001 |


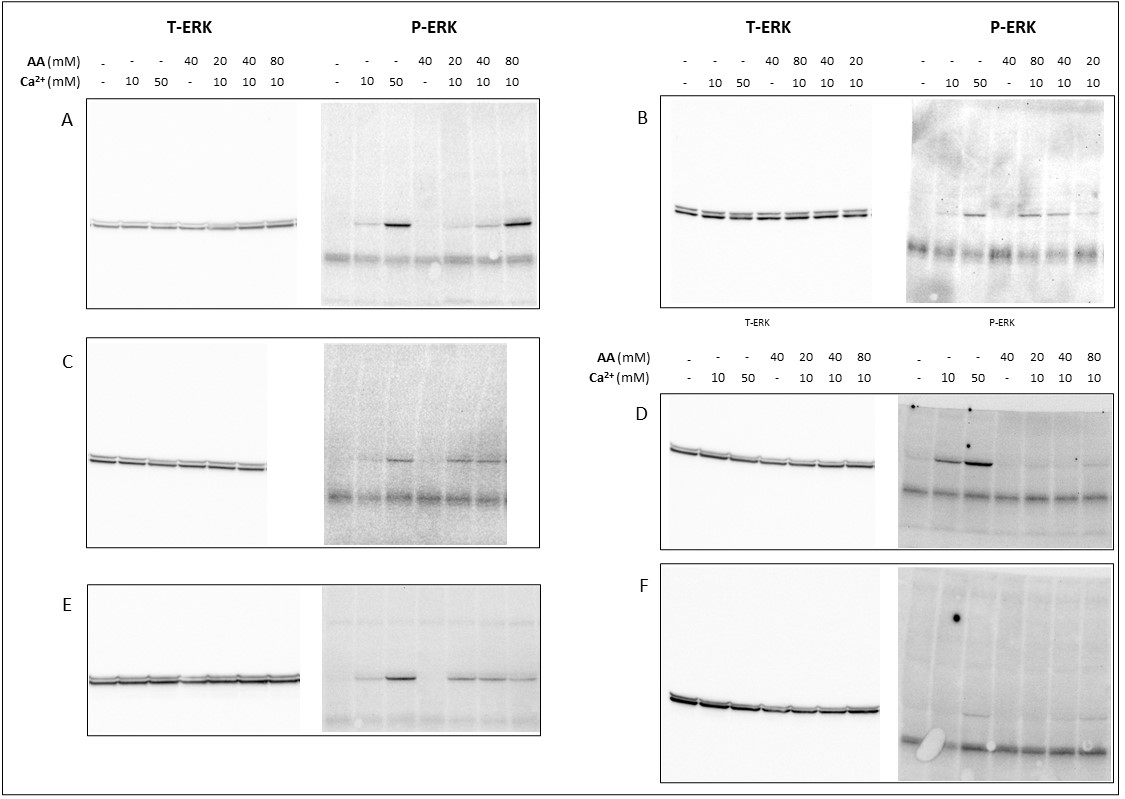


Supplementary Figure S5 Measurements of ERK activation in asCasr-Flp-In-HEK293 cells on stimulation with the amino acids (**A**) L-His, (**B**) L-Phe, (**C**) L-Trp, (**D**) L-Leu, (**E**) L-Ile, and (**F**) L-Val. ERK activation was assessed by Western blotting using antibodies against phosphorylated ERK (P-ERK) and total ERK (T-ERK). Please note that for panel (**B**) the concentration of the amino acid L-Phe is reversed, thus the different legend. The blots are uncropped.

*Note: Supplementary Figure S5 blots overexposed:*


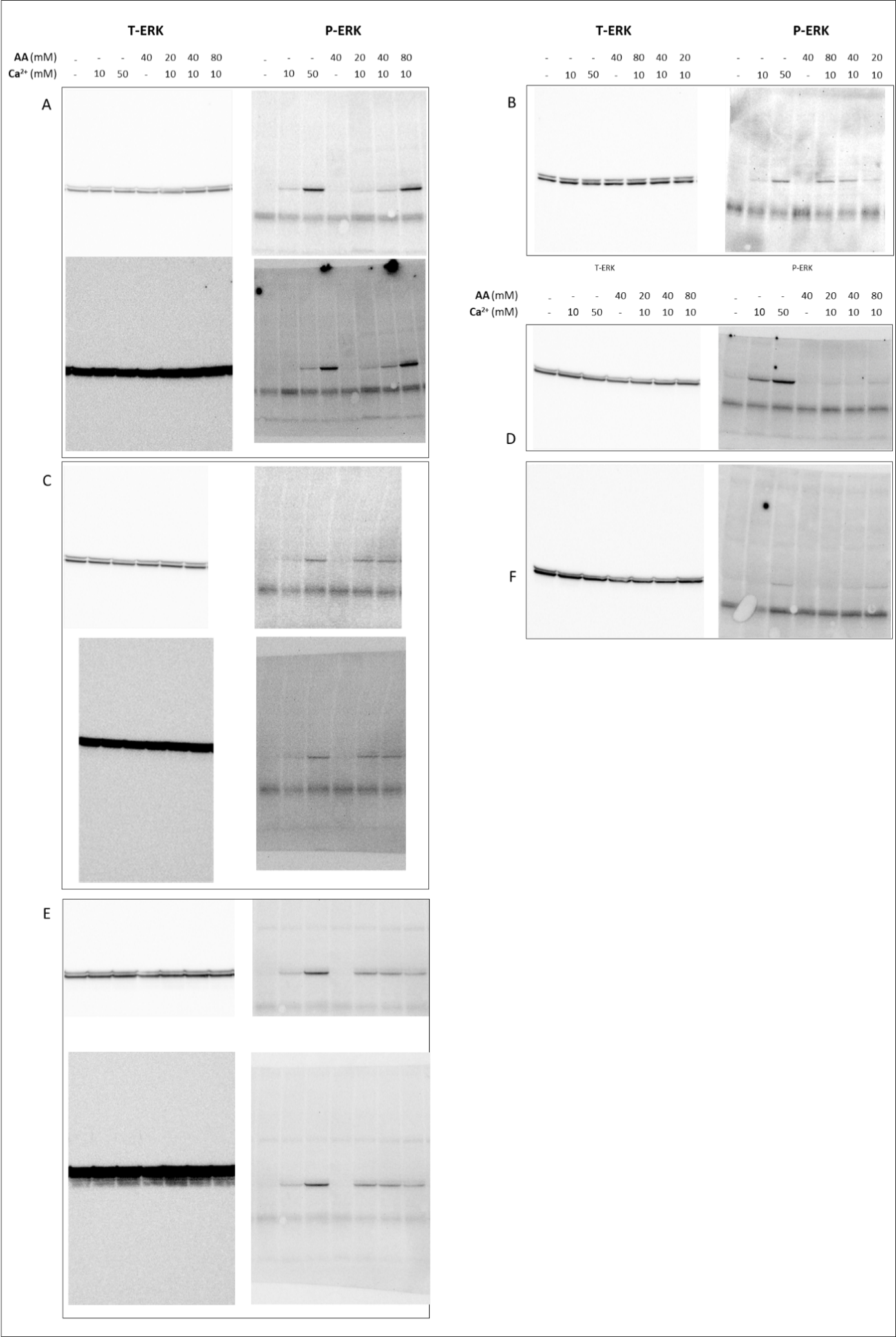


## Multiple alignment of the Atlantic salmon Casr


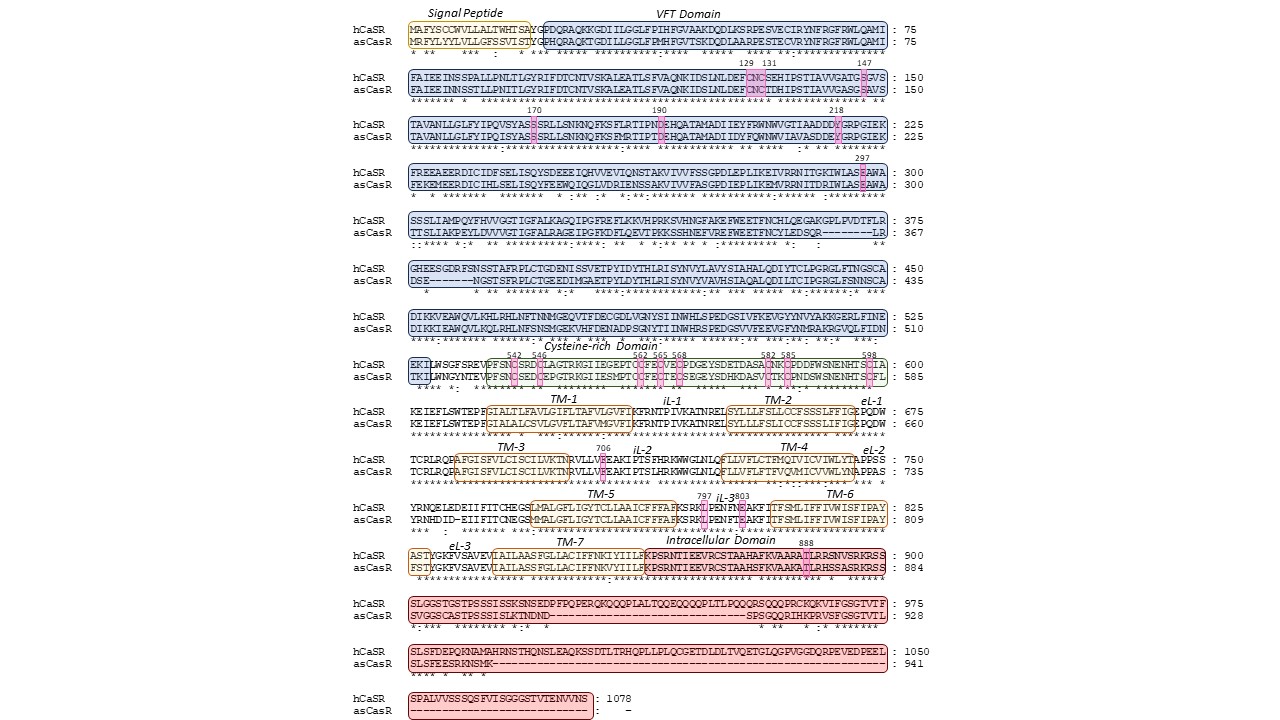


Supplementary Figure S6 Multiple alignment of the Atlantic salmon Casr (asCasr; GenBank Acc. No. NP_001119703.1) and the human CaSR (hCaSR; GenBank Acc. No. P41180) amino acid sequences using ClustalX 2.1 (<https://clustalx.software.informer.com/2.1/>) and edited in GeneDoc 2.7 software (<https://nrbsc.org/gfx/genedoc/>). The annotated features were based on the hCaSR ^1^. The extracellular N-terminal Venus Fly Trap (VFT) is marked with a blue box, and in this domain, the C^129^ and C^131^ that support disulfide linkages between subunits in homodimers is marked with a pink box. The conserved residues involved in amino acid binding are also marked with a pink box (S^147^, S^170^, D^190^, Y^218^ and E^297^). In the Cysteine-rich domain, which transmits signals from the VFT domain, predicted intra-domain disulfides are shown as follows: C^542^–C^562^; C^546^–C^565^; C^568^–C^582^; C^585^–C^598^. The putative transmembrane domains, named TM-1 to TM-7, were drawn using the annotation data of hCaSR ^1^. Residues F^706^ in the intracellular domain 2 (iL-2), and L^797^ and E^803^ in the iL-3 are required for efficient coupling to G_q_. In the intracellular C-terminus, the key phosphorylation site required for the control of the Ca^2+^_i_ mobilizing responses is shown at I^888^ and is also conserved between human and Atlantic salmon sequences. The Atlantic salmon Casr share 69 % of amino acid sequence identity and 78% of similarity with the human CaSR protein, and the most divergent region is the intracellular C-terminal domain of the protein.

## References

1 Conigrave, A. D. & Ward, D. T. Calcium-sensing receptor (CaSR): Pharmacological properties and signaling pathways. *Best Practice & Research Clinical Endocrinology & Metabolism* **27**, 315-331 (2013). https://doi.org:https://doi.org/10.1016/j.beem.2013.05.010
